# Remarkable Anti-*Trichomonas vaginalis* Activity of Plants Traditionally Used by the Mbyá-Guarani Indigenous Group in Brazil

**DOI:** 10.1155/2013/826370

**Published:** 2013-06-20

**Authors:** Clara Lia Costa Brandelli, Patrícia de Brum Vieira, Alexandre José Macedo, Tiana Tasca

**Affiliations:** ^1^Faculdade de Farmácia, Universidade Federal do Rio Grande do Sul, Avenida Ipiranga 2752, 90610-000 Porto Alegre, RS, Brazil; ^2^Faculdade de Farmácia e Centro de Biotecnologia, Universidade Federal do Rio Grande do Sul, Avenida Ipiranga 2752, 90610-000 Porto Alegre, RS, Brazil

## Abstract

*Trichomonas vaginalis*, a flagellate protozoan, is the causative agent of trichomonosis, the most common nonviral sexually transmitted disease worldwide. Taking into account the increased prevalence of metronidazole-resistant isolates, alternative drugs are essential for the successful treatment. Natural products are the source of most new drugs, and popular wisdom about the use of medicinal plants is a powerful tool in this search. In this study, the activity of 10 medicinal plants extensively used in daily life by Mbyá-Guarani indigenous group was evaluated against seven different *T. vaginalis* isolates. Among the aqueous extracts tested, *Verbena* sp. (*Guachu ka'a* in Mbyá-Guarani language) and *Campomanesia xanthocarpa* (*Guavira* in Mbyá-Guarani language) showed the highest activity against *T. vaginalis* with MIC value of 4.0 mg/mL reaching 100% of efficacy against the parasite. The kinetic growth assays showed that the extracts promoted complete growth abolishment after 4 h of incubation. In addition, the extracts tested did not promote a significant hemolytic activity against human erythrocytes. Our results show for the first time the potential activity of *Verbena* sp. and *C. xanthocarpa* against *T. vaginalis*. In addition, this study demonstrates that indigenous knowledge is an important source of new prototype antiprotozoal agents.

## 1. Introduction

*Trichomonas vaginalis* parasitizes the urogenital human tract causing trichomonosis, the most prevalent non-viral sexually transmitted disease worldwide, being responsible for 248 million new cases annually [1]. After colonization, the parasite causes vaginitis, urethritis, and prostatitis [2]. Moreover, the pathogen has been associated with serious consequences as adverse pregnancy outcomes and preterm birth [3], infertility [4], predisposition to cervical cancer [5], and pelvic inflammatory disease [6]. Importantly, trichomonosis acts as a cofactor in human immunodeficiency virus (HIV) transmission and acquisition [7, 8]. In spite of impact of this infection, the therapeutic arsenal is restricting, and only metronidazole and tinidazole, both 5-nitroimidazole drugs, are approved by the FDA for trichomonosis treatment [9]. Although the cure rate is high, treatment failure can be observed and the resistance of *T. vaginalis* isolates is the main reason of treatment failure [10]. In this sense, it is undoubtedly necessary that the investigation for new alternatives for the treatment of trichomonosis and two strategies can be followed: (i) the search for new therapeutic targets, which is essential to rational development of new antiparasitic agents and (ii) the investigation of new anti-*T*. *vaginalis* compounds structurally distinct from 5-nitroimidazoles and, consequently, acting by different mechanism as demonstrated by our group [11, 12].

In this context, natural products, especially medicinal plants, are immeasurable as a potent source of bioactive molecules. Since ancient times, people use plants to treat common infectious diseases, and some of these traditional medicines are still integrated as part of the cure of diverse pathologies [13]. In addition, the search based on ethnopharmacological information rescues the immense empirical knowledge of plants utilization [14, 15]. In this context, regarding natural environment and vast wealth of genetic resources, Brazil is a country of great interest to ethnopharmacology [16].

 Indigenous people have a wealth of biodiversity information and know how to capture and use natural resources [17]. Despite of the fact that several drugs have been discovered through traditional cures and folk knowledge, in Brazil there are 122 indigenous ethnic groups, but only 30% of them were investigated with regard to ethnobotanical aspects [18, 19]. Therefore, the study of plants used by indigenous medicine is important to interconnect traditional medicine and biotic environment preserving the indigenous ancient knowledge.

Parasitic protozoa remain a major threat to human and animal health, and there are few effective drugs for the treatment of many protozoal diseases. Taking into account the indigenous ethnopharmacology as a contributor to the systematic screening of plants with antiprotozoal activity, this study evaluated the anti-*T*. *vaginalis* activity of the most important medicinal plants used in Mbyá-Guarani indigenous medicine located in South of Brazil.

## 2. Materials and Methods

### 2.1. Parasite Culture Conditions

In this study, seven isolates of *T. vaginalis *were used: 30236 and 30238 from American Type Culture Collection (ATCC); TV-LACM1, TV-LACM2, and TV-LACM3 (fresh clinical isolates from female patients); TV-LACH1 and TV-LACH2 (fresh clinical isolates from male patients) all from Laboratório de Análises Clínicas e Toxicológicas, Faculdade de Farmácia, UFRGS, Brazil (project with ethical approval by UFRGS Ethical Research Committee, number 18923). Trichomonads were axenically cultured in trypticase-yeast extract-maltose (TYM) medium (pH 6.0), supplemented with 10% heat-inactivated bovine serum and incubated at 37°C [20].

### 2.2. Plant Material

*Plant material* was collected from indigenous tribe Mbyá-Guarani located at the Lomba do Pinheiro, Porto Alegre, RS, Brazil (30°06′47.62′′ S and 51°07′37.85′′ W), in March 2011. This village is denominated *Tekoá Anhetenguá* (in English, *True Village*), and 200 people live in the area gathered at 40 families. The women of the village have the knowledge of medicinal plants used by the entire population and conducted the collection of specimens. Ten plants routinely used in indigenous medicine for infectious diseases were collected: leaves of *Aloe arborescens *Mill. (ICN 173371), aerial parts of *Bidens pilosa* L. (ICN 167397), aerial parts of *Rhipsalis baccifera *(ICN 167402), barks of *Luehea divaricata *(ICN 167403), roots of *Trichilia *sp. (ICN 173126), leaves of *Campomanesia xanthocarpa* O. Berg. (ICN 167401), leaves of *Coix lacryma-jobi *Lin. (ICN 167396), leaves of *Citrus limonium *(ICN 167399), leaves of *Citrus reticulata* (ICN 173127), and leaves of *Verbena *sp. (ICN 167394). The voucher specimens were deposited at the herbarium of the Universidade Federal do Rio Grande do Sul (ICN).

### 2.3. Preparation of Plant Extracts

The fresh plants extracts were prepared conforming traditional use by indigenous people by decoction at 60°C for 60 min [1 : 10; (w : v)]. Aqueous extracts were freeze dried, and the work solution was prepared at 8.0 mg/mL in ultrapure water, sterilized by filtration (0.22 *µ*m) and stored at −20°C.

### 2.4. Anti-*Trichomonas vaginalis* Assay

Ten aqueous extracts were screened *in vitro* for activity against *T. vaginalis*. The assay was performed in 96 microtiter plates at final concentration of 4.0 mg/mL in the wells. The inhibitory minimum concentration (MIC) was determined using an eightfold dilution (4.0–0.031 mg/mL), and the viability of the trophozoites was determined according to a fluorimetric method [21]. Two controls were performed: negative control only with trophozoites and a positive control with 8.0 *µ*M metronidazole (Sigma Chemical Co., St. Louis, MO, USA). The experiments were performed at least in three independent experiments, in triplicate. The results were expressed as the percentage of viable trophozoites compared to untreated parasites.

### 2.5. Growth Effect of Aqueous Extracts

In order to investigate the effect of the active aqueous extracts on *T. vaginalis* growth, a kinetic growth curve was performed using the ATCC 30236 isolate. The parasites initial density of 2.0 × 10^5^ trophozoites/mL was incubated with the extracts at MIC value in TYM medium. The trophozoites were counted using a hemocytometer during 72 h. The results were expressed as trophozoite number (10^5^/mL). For microscopic analysis, the controls and treated parasites were centrifuged for 10 min at 2,000 rpm to concentrate the organisms after 4 h of incubation, and the preparation was observed in light microscope (magnified ×1,000) by trypan blue dye exclusion.

### 2.6. Hemolytic Assay

This assay was performed according to the method used by Rocha et al. [22]. Blood type O positive from healthy human volunteers was collected with Alsever's solution (1 : 1) and centrifuged at 2,000 rpm for 5 min. The erythrocyte fraction was washed three times with PBS 1x (pH 7.0) and resuspended to give 1% red cell suspension (v/v). Aqueous extracts concentrations were chosen according to minimum inhibitory concentration (MIC) determined in the susceptibility assay. The erythrocytes were incubated with the extracts at 37°C under agitation for 1 h and then were centrifuged at 3,000 rpm for 5 min. The absorbance of the supernatant was measured at 540 nm. The experiment was performed in triplicate, and the percentage of hemolysis caused by each sample tested was calculated in comparison to 100% hemolytic activity of saponin from *Quillaja saponaria* [23].

## 3. Results and Discussion

**Figure 1:**
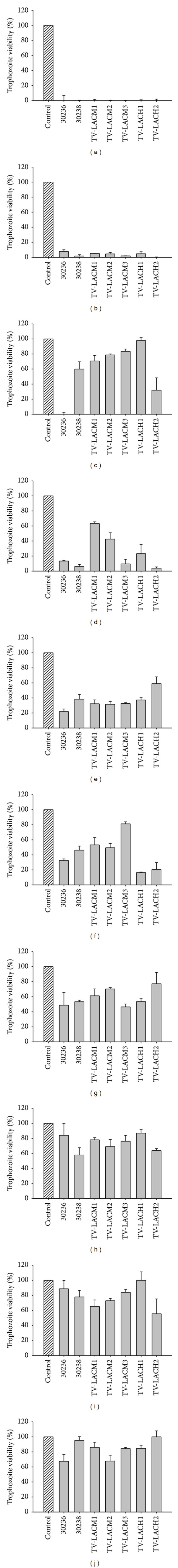
Effect of selected Mbyá-Guarani medicinal plants at 4.0 mg/mL on different *Trichomonas vaginalis* isolates. (a) *Verbena *sp. (b)* Campomanesia xanthocarpa*. (c) *Bidens pilosa.* (d) *Luehea divaricata.* (e) *Coix lacryma-jobi*. (f) *Trichilia *sp. (g) *Citrus limonium. *(h) *Aloe arborescens. *(i) *Citrus reticulata*. (j) *Rhipsalis baccifera*. Data represent means ± standard deviation.

This study evaluated the anti-*T. vaginalis* activity of aqueous extracts of 10 medicinal plants used in medicine system Mbyá-Guarani. Importantly, among the plants tested, we found two aqueous extracts with a potent anti-*T. vaginalis in vitro* activity. The promising extracts are of *Verbena *sp. and *Campomanesia xanthocarpa. *The extract from *Verbena *sp., at 4.0 mg/mL, demonstrated the optimal anti-*T. vaginalis *activity inducing complete cytotoxicity (100% of parasites death) upon all distinct trichomonads isolates (Figure 1(a)). The leaves extract of *Campomanesia xanthocarpa *at the same concentration also showed a very high anti-*T. vaginalis* activity, reducing the parasite viability up to 96% (Figure 1(b)). 

Other aqueous extracts displayed a variety of results. The aqueous extract obtained from *Bidens pilosa* totally abolished the trophozoites viability only of the 30236 isolate (Figure 1(c)). The extract of the barks of *Luehea divaricata* induced a reduction of *T. vaginalis* viability differently in each trichomonads isolate tested (Figure 1(d)). The leaves of *Coix lacryma-jobi* induced, on average, 75% of viability reduction on *T. vaginalis* isolates; however, for the TV-LACH2 isolate, the reduction was only 40% (Figure 1(e)). The fresh clinical isolates from male patients (TV-LACH1 and TV-LACH2) presented higher susceptibility than the isolates from female patients (ATCC and fresh clinical) when the root extract of *Trichilia* sp. was tested (Figure 1(f)). Extracts of leaves from *Citrus limonium* reduced about 40% the viability of isolates (Figure 1(g)). The extracts from *Aloe arborescens* and *Citrus reticulata* decreased the viability of 50% of at least one isolate (Figures 1(h) and 1(i)), while *Rhipsalis baccifera* reduced only 30% the viability of 30236 and TV-LACM2 isolates (Figure 1(j)).

In comparison to the other extracts, the extracts from *Verbena* sp. and *C. xanthocarpa* demonstrated the most promising activity against *T. vaginalis,* and the minimum inhibitory concentration was determined for the 30236 isolate (Table 1).

**Figure 2:**
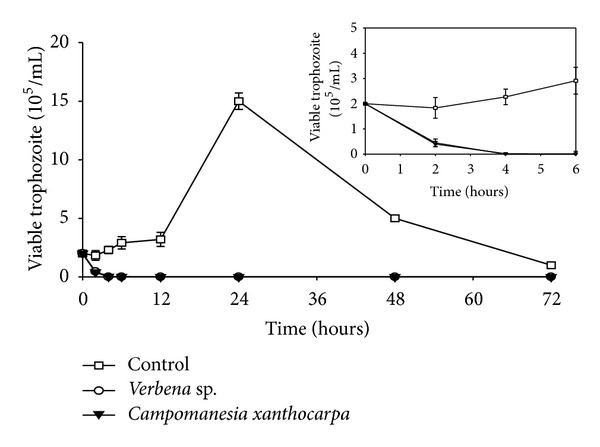
Kinetic growth curve of *Trichomonas vaginalis* 30236 isolate after treatment with aqueous extracts of *Verbena* sp. and *Campomanesia xanthocarpa* at 4.0 mg/mL. The trophozoites growth was completely inhibited by the extracts in 4 hours of incubation. Data represent means ± standard deviation of at least three experiments, all in triplicate.

**Figure 3:**
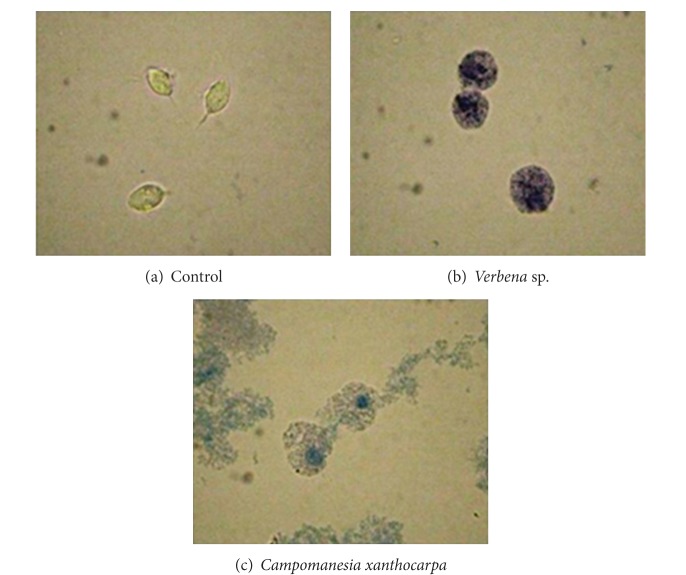
Microscopic analysis of parasites morphology in light microscope (magnified ×1,000) by trypan blue dye exclusion. (a) Control; (b) after 4 hours of treatment with aqueous extract of *Verbena* sp.; (c) after 4 hours of treatment with aqueous extract of *Campomanesia xanthocarpa*.

In order to investigate the effect of the most active extract on parasite growth and viability, a kinetic growth curve was performed. The control exhibited the classical growth peak after 24 h of incubation; however, the organisms treated with extracts of *Verbena* sp. and *C. xanthocarpa*, after 4 h of incubation, evoked a complete growth abolishment (Figure 2) and showed a characteristic blue color by trypan blue dye exclusion indicating cell death (Figure 3). 

In order to investigate a possible effect of the *C. xanthocarpa* and *Verbena* sp. extracts on parasites membrane and also to have preliminary data on cytotoxicity against mammalian cells, hemolytic activity of these extracts was conducted. The *Verbena* sp. and *C. xanthocarpa* extracts were tested at MIC values and demonstrated 6.5 and 7.6% erythrocytes lysis, respectively (Table 1). These results demonstrated that both extracts, at MIC values, did not promote a significant hemolytic activity, indicating that the mechanism of action responsible for the parasite viability reduction probably does not involve parasitic membrane disruption. Moreover and importantly, these findings suggest that both extracts are not toxic to mammalian cells, since they did not lyse human erythrocytes.

This study contributes to rescue the indigenous knowledge about medicinal plants. The indigenous Mbyá-Guarani use *Verbena* sp. (*Guachu ka'a* in Mbyá-Guarani language), as tea, for relief of symptoms of infectious diseases, such as fever. This is in agreement of the literature that relates *Verbena* sp. infusion as sedative, analgesic to inflammatory disorders, skin burns, amenorrhea, depression, and gastric diseases [24–26]. Moreover, this species has a variety of chemical constituents, as flavonoid, iridoid glycosides, phenylpropanoid glycoside, sterols, triterpenes, and glycoconjugate, and the biological activity may be partially attributed to them [24]. In addition, this tribe utilizes the leaves of *C. xanthocarpa* (*Guavira* in Mbyá-Guarani language) for treatment of diarrhea, stomach pain, and worms. This plant is traditionally used in the south of Brazil as depurative, antidiarrhoeic, cleanser, and antirheumatic, and to decrease the blood cholesterol [27], and these properties may be attributed to tannins, saponins, and flavonoids (quercetin, myricetin, quercitrin, and rutin), compounds present in the leaves of *C. xanthocarpa* [28].

The rescue ethnopharmacology of historical uses of plants is recognized as valuable for bioprospection, since they afford the rationale for selection and research of medicinal plants [15]. This study describes the first report on the antiprotozoal activity of *Verbena* sp. and *C. xanthocarpa*. In addition, other species of the families Verbenaceae and Myrtaceae were tested against *T. vaginalis*, however, without success [29, 30]. Several biodiversities and traditional knowledge of different populations have guided studies to search new prototypes anti-*T. vaginalis*. Recently, the potential of plants traditionally used from Caatinga against *T. vaginalis* was reported [31]. Calzada et al. [29] demonstrated that plants used for urogenital tract disorders in Mexican traditional medicine showed effect against this protozoan. Amaryllidaceae species used against venereal diseases in South Africa also showed promising activity against *T. vaginalis* [11, 32]. 

Considering the impact of trichomonosis in public health and the emergent number of resistant *T. vaginalis* isolates, it is necessary alternatives for the treatment of this infection. Natural products are a promising source of active molecules, and the ethnopharmacology approach rescues the knowledge of population of medicinal plants. This wisdom combined with chemical and pharmacological studies presents a precious value in the bioprospection of innovates, safe, and accessible drugs. This pioneer study demonstrated relevant results about anti-*T. vaginalis* activity of the *Verbena* sp. and *C. xanthocarpa*, plants traditionally used by indigenous population Mbyá-Guarani for infectious diseases. Despite of showing strong anti-*T. vaginalis* activity, this report rescued the knowledge of indigenous people, avoiding the miscarriage of this wisdom.

## Figures and Tables

**Table 1:** Hemolytic activity of the aqueous extracts that showed the best anti-*Trichomonas vaginalis* activity and respective MIC determined by susceptibility assay.

	Aqueous extract	
	*Verbena* sp.	*Campomanesia xanthocarpa *
MIC (mg/mL)	4.0	4.0
Hemolysis (%) ± S.D.	6.5 ± 0.02	7.6 ± 0.01
